# The common HAQ STING variant impairs cGAS-dependent antibacterial responses and is associated with susceptibility to Legionnaires’ disease in humans

**DOI:** 10.1371/journal.ppat.1006829

**Published:** 2018-01-03

**Authors:** Juan S. Ruiz-Moreno, Lutz Hamann, Javeed A. Shah, Annelies Verbon, Frank P. Mockenhaupt, Monika Puzianowska-Kuznicka, Jan Naujoks, Leif E. Sander, Martin Witzenrath, John C. Cambier, Norbert Suttorp, Ralf R. Schumann, Lei Jin, Thomas R. Hawn, Bastian Opitz, CAPNETZ Study Group

**Affiliations:** 1 Department of Internal Medicine/Infectious Diseases and Pulmonary Medicine, Charité - Universitätsmedizin Berlin, corporate member of Freie Universität Berlin, Humboldt-Universität zu Berlin, and Berlin Institute of Health, Berlin, Germany; 2 Institute of Microbiology and Hygiene, Charité - Universitätsmedizin Berlin, corporate member of Freie Universität Berlin, Humboldt-Universität zu Berlin, and Berlin Institute of Health Berlin, Berlin, Germany; 3 Department of Medicine, University of Washington, Seattle, Washington, United states of America; 4 VA Puget Sound Health Care System, Seattle, Washington, United states of America; 5 Department of Medical Microbiology and Infectious diseases, Erasmus University Medical Center, Rotterdam, The Netherlands; 6 Institute of Tropical Medicine and International Health, Charité - Universitätsmedizin Berlin, corporate member of Freie Universität Berlin, Humboldt-Universität zu Berlin, and Berlin Institute of Health, Berlin, Germany; 7 Department of Human Epigenetics, Mossakowski Medical Research Centre, Polish Academy of Sciences, Warsaw, Poland; 8 Department of Geriatrics and Gerontology, Medical Centre of Postgraduate Education, Warsaw, Poland; 9 German Center for Lung Research (DZL), Germany; 10 CAPNETZ STIFTUNG, Hannover, Germany; 11 Department of Immunology and Microbiology, University of Colorado School of Medicine, Aurora, Colorado, United States of America; 12 Department of Medicine, Division of Pulmonary, Critical Care and Sleep Medicine, University of Florida, Gainesville, Florida, United States of America; University of São Paulo FMRP/USP, BRAZIL

## Abstract

The cyclic GMP-AMP synthase (cGAS)-STING pathway is central for innate immune sensing of various bacterial, viral and protozoal infections. Recent studies identified the common HAQ and R232H alleles of *TMEM173/STING*, but the functional consequences of these variants for primary infections are unknown. Here we demonstrate that cGAS- and STING-deficient murine macrophages as well as human cells of individuals carrying HAQ *TMEM173/STING* were severely impaired in producing type I IFNs and pro-inflammatory cytokines in response to *Legionella pneumophila*, bacterial DNA or cyclic dinucleotides (CDNs). In contrast, R232H attenuated cytokine production only following stimulation with bacterial CDN, but not in response to *L*. *pneumophila* or DNA. In a mouse model of Legionnaires’ disease, cGAS- and STING-deficient animals exhibited higher bacterial loads as compared to wild-type mice. Moreover, the haplotype frequency of HAQ *TMEM173/STING*, but not of R232H *TMEM173/STING*, was increased in two independent cohorts of human Legionnaires’ disease patients as compared to healthy controls. Our study reveals that the cGAS-STING cascade contributes to antibacterial defense against *L*. *pneumophila* in mice and men, and provides important insight into how the common HAQ *TMEM173/STING* variant affects antimicrobial immune responses and susceptibility to infection.

**Trial registration:**

ClinicalTrials.gov DRKS00005274, German Clinical Trials Register

## Author summary

Interferons (IFNs) and pro-inflammatory cytokines are key regulators of gene expression and antibacterial defense during *Legionella pneumophila* infection. Here we demonstrate that production of these mediators was largely or partly dependent on the cyclic GMP-AMP synthase (cGAS)-STING pathway in human and murine cells. Cells of individuals carrying the common HAQ allele of *TMEM173/STING* were strongly impaired in their ability to respond to *L*. *pneumophila*, bacterial DNA or cyclic dinucleotides (CDNs), whereas the R232H allele was only attenuated in sensing of exogenous CDNs. Importantly, cGAS and STING contributed to antibacterial defense in mice during *L*. *pneumophila* lung infection, and the allele frequency of HAQ *TMEM173/STING*, but not of R232H *TMEM173/STING*, was increased in two independent cohorts of human Legionnaires’ disease patients as compared to healthy controls. Hence, sensing of bacterial DNA by the cGAS/STING pathway contributes to antibacterial defense against *L*. *pneumophila* infection, and the hypomorphic variant HAQ *TMEM173/STING* is associated with increased susceptibility to Legionnaires’ disease in humans.

## Introduction

*Legionella pneumophila* is increasingly recognized as a significant cause of pneumonia in ambulatory and hospitalized patients. This form of pneumonia, commonly referred to as Legionnaires’ disease, is associated with high mortality rates, ranging from 8 to 34% depending on the study, despite availability of efficient antibiotic therapies [1]. Known risk factors for Legionnaires’ disease include chronic respiratory and cardiovascular diseases, diabetes, cancer, and immunosuppression, although individuals without these predisposing conditions are also affected by Legionnaires’ disease [1,2]. Infection occurs following inhalation of *L*. *pneumophila*-contaminated water droplets. Once in the alveolar compartment, the bacterium is phagocytosed by alveolar macrophages, where it establishes an intracellular replication vacuole. This process requires the Dot/Icm type IV secretion system (T4SS) which injects approx. 300 bacterial effector molecules into the host cytosol [3].

The immune response to *L*. *pneumophila* in the lung is largely dependent on production of pro-inflammatory cytokines and interferons (IFNs) [4–9]. While IL-1β and TNFα stimulate antibacterial defense by *e*.*g*. promoting neutrophil recruitment [10,11], type I and II IFNs activate an IRG1- and itaconic acid-dependent macrophage-intrinsic resistance pathway [12]. Infected and bystander macrophages are the main producers of IL-1β, type I IFNs and TNFα, respectively [4,13–15], whereas type II IFN is released by innate and adaptive lymphoid cells [16]. The *L*. *pneumophila*-induced type I IFN production has previously been shown to depend on the T4SS and cytosolic sensing of bacterial DNA [4,8,13], although detection of the bacterial cyclic dinucleotides (CDNs) cyclic 3′-5′ diguanylate (c-diGMP) has also been implicated in this response [17]. Moreover, we recently showed that inhibition of the endoplasmic reticulum-associated protein STING (stimulator of IFN genes, also known as MITA, ERIS, MPYS) reduced type I IFN responses to *L*. *pneumophila* in murine macrophages [4].

STING is encoded by the *TMEM173/STING* gene and functions as both a signaling adaptor in the cytosolic DNA sensing pathway [18,19] and as a receptor for bacterial and endogenous CDNs [20]. Upstream of STING, sensing of DNA in the cytosol additionally requires the cyclic GMP-AMP synthase (cGAS), which binds microbial and host DNA and mediates production of the second messenger cyclic 2’3’-GMP-AMP (2’3’-cGAMP) [21,22]. Recent studies have shown that production of type I IFNs during infections with several bacterial pathogens requires both, cGAS and STING [23–26].

Interestingly, human *TMEM173/STING* exhibits significant heterogeneity [27,28]. The second most common allele besides the WT allele is HAQ, which contains a haplotype comprised of the three non-synonymous single nucleotide polymorphisms (SNPs) R71H-G230A-R293Q. Previous studies indicated that HAQ STING is a loss-of-function variant exhibiting largely reduced capacity to stimulate type I IFN responses [27–29]. Moreover, the third most common allele, R232H, has been found to be defective in sensing bacterial CDNs but not of 2’3’-cGAMP or DNA [28,30]. However, the function of endogenous HAQ and R232H STING in primary infections and the effect of these variants on susceptibility to diseases in humans have not yet been addressed.

Here we tested the hypotheses that i) the cGAS/STING pathway mediates defense against *L*. *pneumophila* in mice and men, ii) that carriage of HAQ *TMEM173/STING* impairs the antibacterial immune response, and iii) that carriage of HAQ *TMEM173/STING* predisposes to Legionnaires’ Disease.

## Results

### *L*. *pneumophila* infection stimulates type I IFN responses *in vitro* and *in vivo* via cGAS and STING

**Fig 1:**
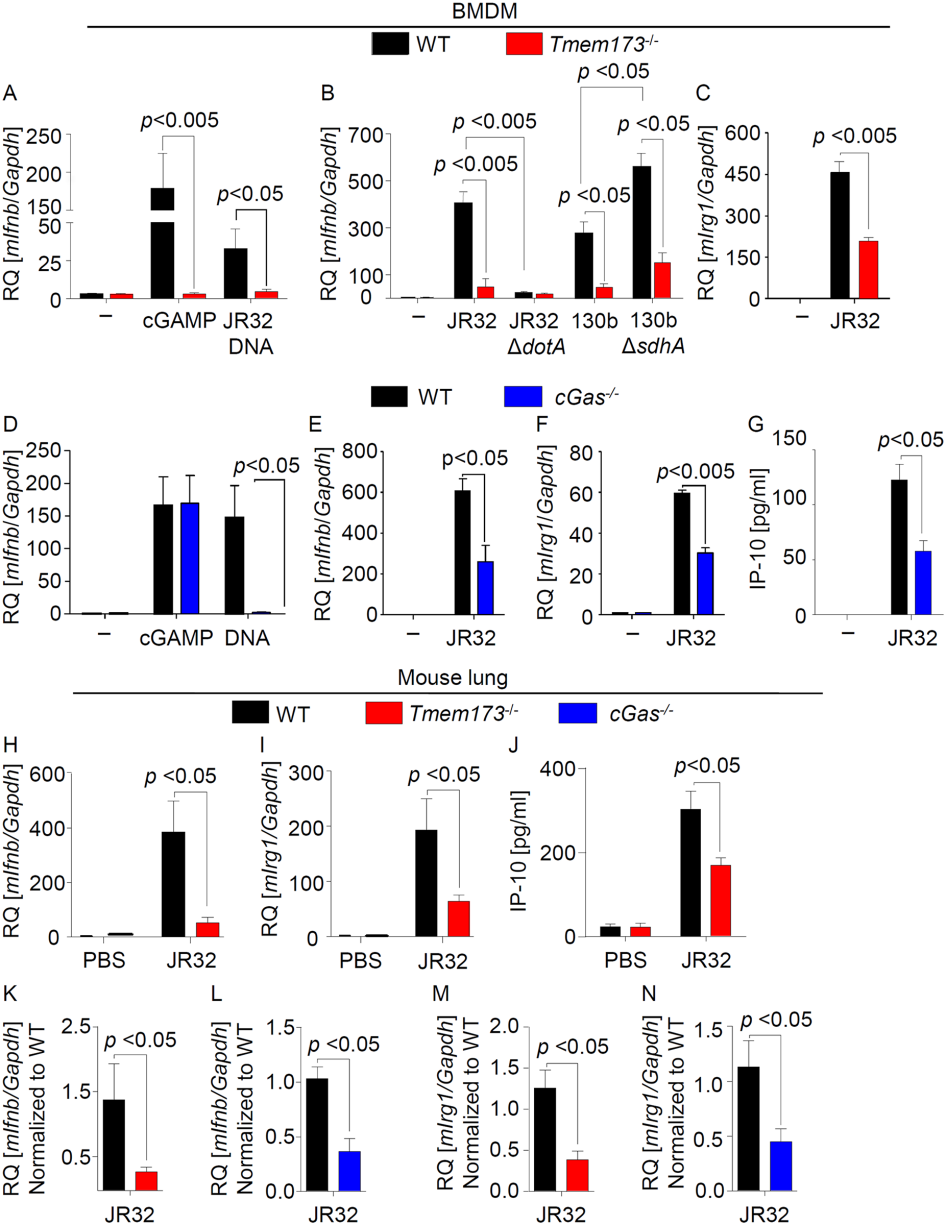
Type I IFN responses during *L*. *pneumophila* infection are mediated by the cGAS/STING pathway. (A-C) WT and *Tmem173*^-/-^ mouse BMDMs were left untreated or stimulated with 1 ug/ml *L*. *pneumophila* DNA (JR32 DNA) or 5 ug/ml 2´3-cGAMP (A) or were infected with *L*. *pneumophila* JR32 WT and 130b WT, or mutant strains deficient for *dotA* or *sdhA* at MOI 10 for 6 h (B, C). Expression of *Ifnb* (A, B) or *Irg1* (C) was measured by qRT-PCR. (D-G) WT and cGAS-deficient BMDMs were stimulated with *L*. *pneumophila* DNA or 2´3-cGAMP or infected with *L*. *pneumophila* JR32 WT, and expression of *Ifnb* and *Irg1* was quantified by qRT-PCR (D-F) or production of IP-10 was measured by ELISA (G). (H-N) WT, STING- and cGAS-deficient mice were intranasally infected with 1×10^6^ *L*. *pneumophila* JR32 WT or instilled with PBS as control (H-J). *Ifnb* and *Irg1* expression in the lungs was assessed 48 (H, I) or 144 h p.i. (K-N) by qRT-PCR, or IP-10 production was measured at 48 h (J). Data are represented as the relative quantification (RQ) of specified mRNAs. Data are shown as the mean + SEM of three to four independent experiments, measured in technical duplicates (Fig. 1A-G) or 6 to 7 mice per group (Fig. H-N). Analyses were performed through the Mann-Whitney U Test. Comparisons with a *p* < 0.05 were considered significant.

Given the critical role of type I IFNs during *L*. *pneumophila* infection, we first investigated the role of STING in inducing type I IFN responses in response to *L*. *pneumophila* as well as *Legionella* DNA in murine bone marrow-derived macrophages (BMDMs). A strong *Ifnb* induction was observed in WT but not in STING-deficient cells in response to two different *L*. *pneumophila* strains, bacterial DNA as well as our control treatment cGAMP (Fig 1A and 1B). The uptake of *L*. *pneumophila* into *Tmem173*^-/-^ BMDMs or its replication was, however, not significantly different as compared to WT cells (S1A Fig). As previously shown, *Legionella* lacking the T4SS effector protein sdhA (*ΔsdhA*) [31], which is involved in maintaining the integrity of the *Legionella*-containing vacuole, induced a stronger type I IFN response, whereas a mutant lacking an essential component of the T4SS (*ΔdotA*) was unable to activate STING-dependent *Ifnb* expression (Fig 1B). Moreover, *L*. *pneumophila*-induced expression of the IFN-stimulated gene *Irg1* and production of the IFN-stimulated chemokine IP-10 were also diminished in *Tmem173*^-/-^ cells (Fig 1C, S1B Fig).

In order to examine the effect of cGAS, BMDMs were first transfected with a siRNA targeting *cGas* or a control siRNA. *cGas*-specific siRNA reduced the expression of its target gene (S2A Fig), and strongly diminished the induction of *Ifnb* and the IFN-induced gene *Irg1* in response to *L*. *pneumophila* or bacterial DNA (S2B and S2C Fig). As expected, cGAMP-stimulated *Ifnb* expression was not inhibited by *cGas* siRNA, since cGAMP is the second messenger produced downstream of cGAS [21,22]. To further demonstrate the importance of cGAS, we challenged murine cGAS-deficient BMDMs with *L*. *pneumophila*, DNA and cGAMP. Importantly, *L*. *pneumophila*- and DNA-induced (but not cGAMP-stimulated) *Ifnb* and *Irg1* expression as well as IP-10 production were reduced in cGAS-deficient BMDMs as compared to WT cells (Fig 1D–1G).

To investigate the relevance of the cGAS/STING pathway for the type I IFN response during lung infection, we intranasally infected WT and *Tmem173*^-/-^ animals with *L*. *pneumophila*. We observed a strongly decreased induction of *Ifnb* and *Irg1* expression as well as IP-10 production in the lungs of STING-deficient mice 48 h p.i (Fig 1H–1J). Similarly, we found significantly reduced expression levels of *Ifnb* and *Irg1* in lung homogenates from cGAS- and STING-deficient mice 144 h p.i. (Fig 1K–1N). In conclusion, our results show that the cGAS/STING pathway is largely responsible for type I IFN responses to *L*. *pneumophila* infection in mice.

### Production of pro-inflammatory cytokines in response to *L*. *pneumophila* is partly dependent on cGAS/STING

**Fig 2:**
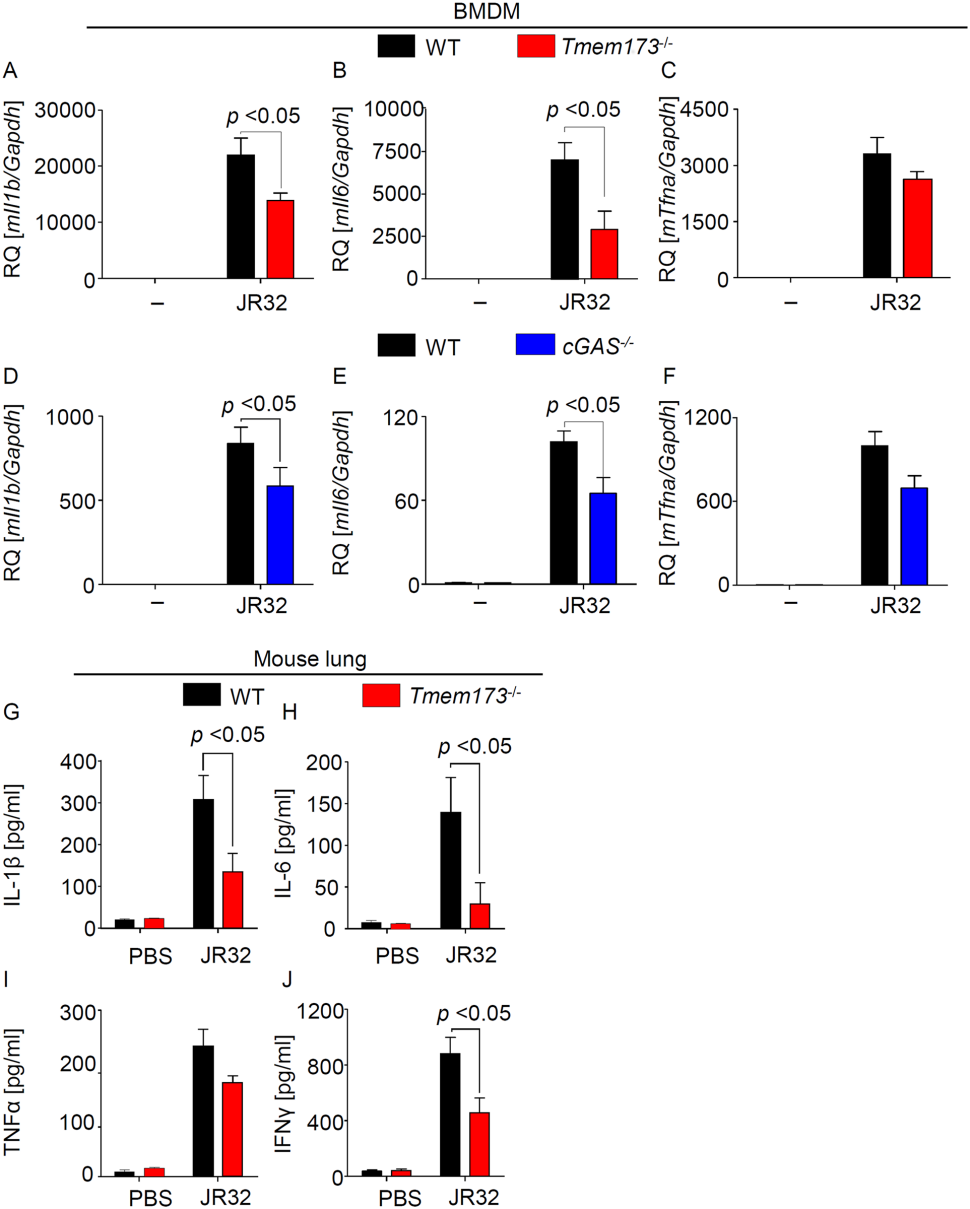
The cGAS/STING axis contributes to the production of pro-inflammatory cytokines during *L*. *pneumophila* infection. (A-F) WT, *Tmem173*^*-/-*^ *and cGas*^*-/-*^ BMDMs were infected for 6 h with *L*. *pneumophila* WT at MOI 10 and relative cytokine expression was determined by qRT-PCR. (G-J) Cytokine protein concentrations in whole lung homogenates from *L*. *pneumophila*-infected mice were quantified by sandwich ELISA. Data are shown as mean ± SEM. (A-F) Data representative of 3 to 4 independent experiments carried out in duplicates. (G-J) Data representative of 6 o 7 mice per group. Data were analyzed through the Mann-Whitney U Test. Comparisons with a *p* < 0.05 were considered significant.

Next we examined the impact of the cGAS-STING pathway on the production of other pro-inflammatory cytokines in response to *L*. *pneumophila*. Deficiency of STING or cGAS significantly reduced production of IL-1β and IL-6 and additionally showed some minor effects on TNFα (Fig 2A–2F, S3A–S3C Fig). Moreover, STING-deficient animals produced less IL-1β and IL-6 as well as IFNγ in response to *L*. *pneumophila* infection of the lung (Fig 2G, 2H and 2J), whereas the effect of STING on TNFα production *in vivo* was not significant (Fig 2I). These data indicate that the cGAS/STING pathway also contributes to the production of pro-inflammatory mediators during *Legionella* infection.

### Endogenous HAQ STING is strongly impaired, but not deficient, in mediating type I IFN and pro-inflammatory cytokine responses to *Legionella* infection or stimulation with DNA and cGAMP

**Fig 3:**
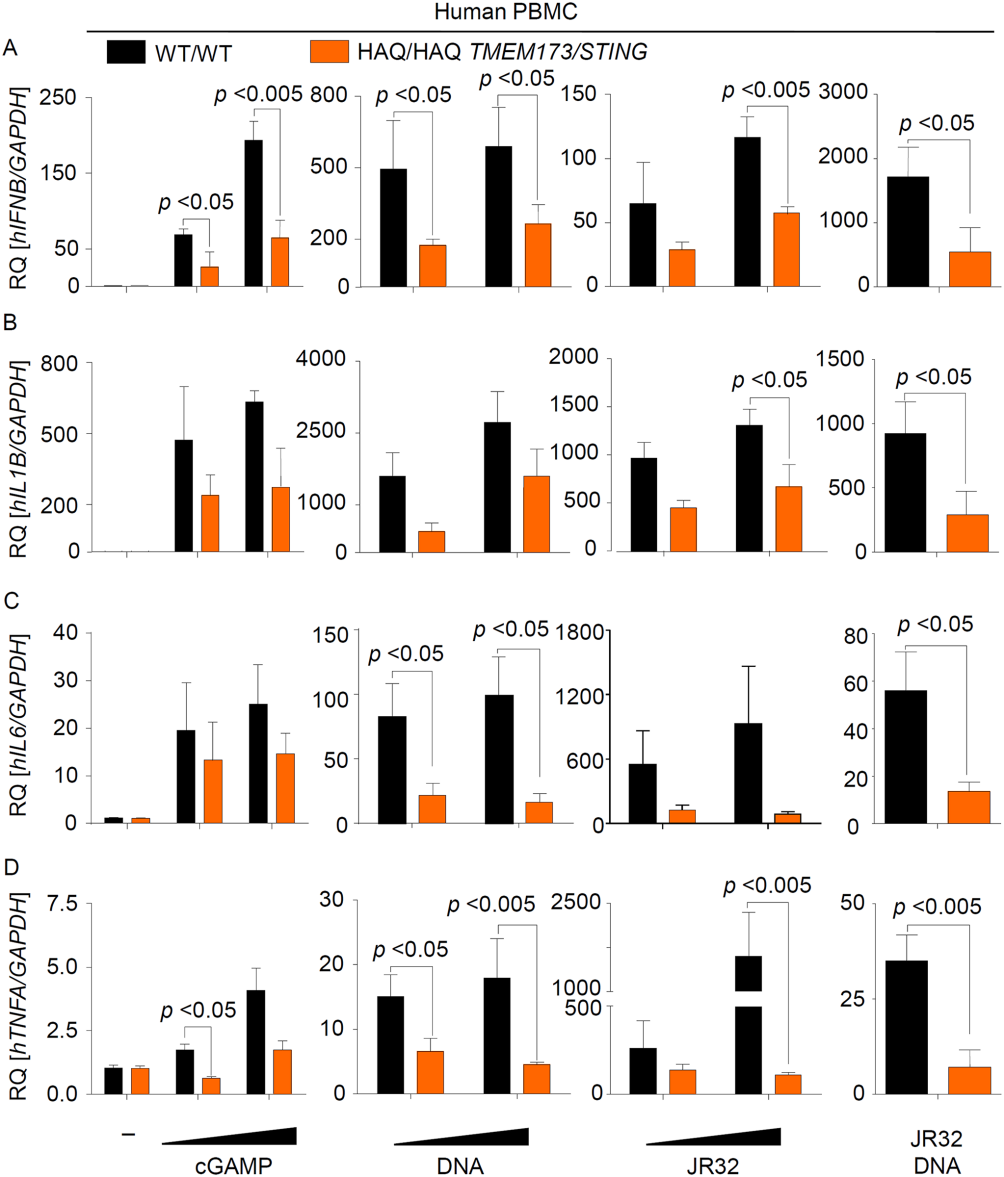
Endogenous HAQ STING is strongly impaired in mounting a type I IFN and proinflammatory cytokine responses against *Legionella* infection or stimulation with DNA or CDNs. (A-D) PBMCs from healthy volunteers (N = 4 for WT and N = 4 for HAQ) were isolated by density gradient centrifugation. 7 d after isolation cells were infected for 6 h with *L*. *pneumophila* at MOIs 10 and 50 or stimulated for the same period with 1 and 5 ug/ml 2´-3´cGAMP or either bacterial or synthetic DNA at a concentration of 0.2 or 1 ug/ml. RNA was isolated and the expression of *IFNB* (A), *IL1B* (B), *IL6* (C) and *TNFA* (D) was determined by qRT-PCR. Data are shown as the RQ of specified mRNAs. Data represent the mean ± SEM of 4 independent experiments carried out in triplicates. Differences were assessed with the Mann-Whitney U Test. Comparisons with a p < 0.05 were considered significant.

Recent studies showed that HAQ STING poorly activates type I IFN responses when ectopically expressed in HEK293 cells [27,28]. In order to examine the activity of endogenous human HAQ STING, we screened about 564 healthy volunteers for the presence of HAQ *TMEM173/STING*. We identified 8 individuals who were homozygous for HAQ (and R232), isolated peripheral blood mononuclear cells (PBMCs) from 4 of them, cultured the cells for 7 days to let the monocytes differentiate into macrophage-like cells, and compared them with cells from persons carrying WT *TMEM173/STING*. In line with our results from STING-deficient murine macrophages (see S1 Fig), we found that replication of *L*. *pneumophila* was not different in cells expressing WT or HAQ STING (S4 Fig). Interestingly, however, we observed a strong reduction in *Ifnb* expression and in production of the IFN-dependent cytokine IP-10 in cells from homozygous HAQ *TMEM173/STING* carriers as compared to cells from WT allele carriers in response to cGAMP, synthetic DNA, *Legionella* infection, and bacterial DNA, but not following stimulation with the TLR7/8 agonist Resiquimod (R848) (Fig 3A, S5A and S6 Figs). HAQ PBMCs were also partly defective in producing pro-inflammatory cytokines such as IL-1β, IL-6 and TNFα (Fig 3B–3D, S5B–S5D Fig). Moreover, heterozygous carriage of HAQ *TMEM173/STING* also lead to a partial reduction of type I IFN and IL-1β expression, which however only reached statistical significance for cGAMP- and *L*. *pneumophila*-induced *IFNB* induction (S7 Fig).

**Fig 4:**
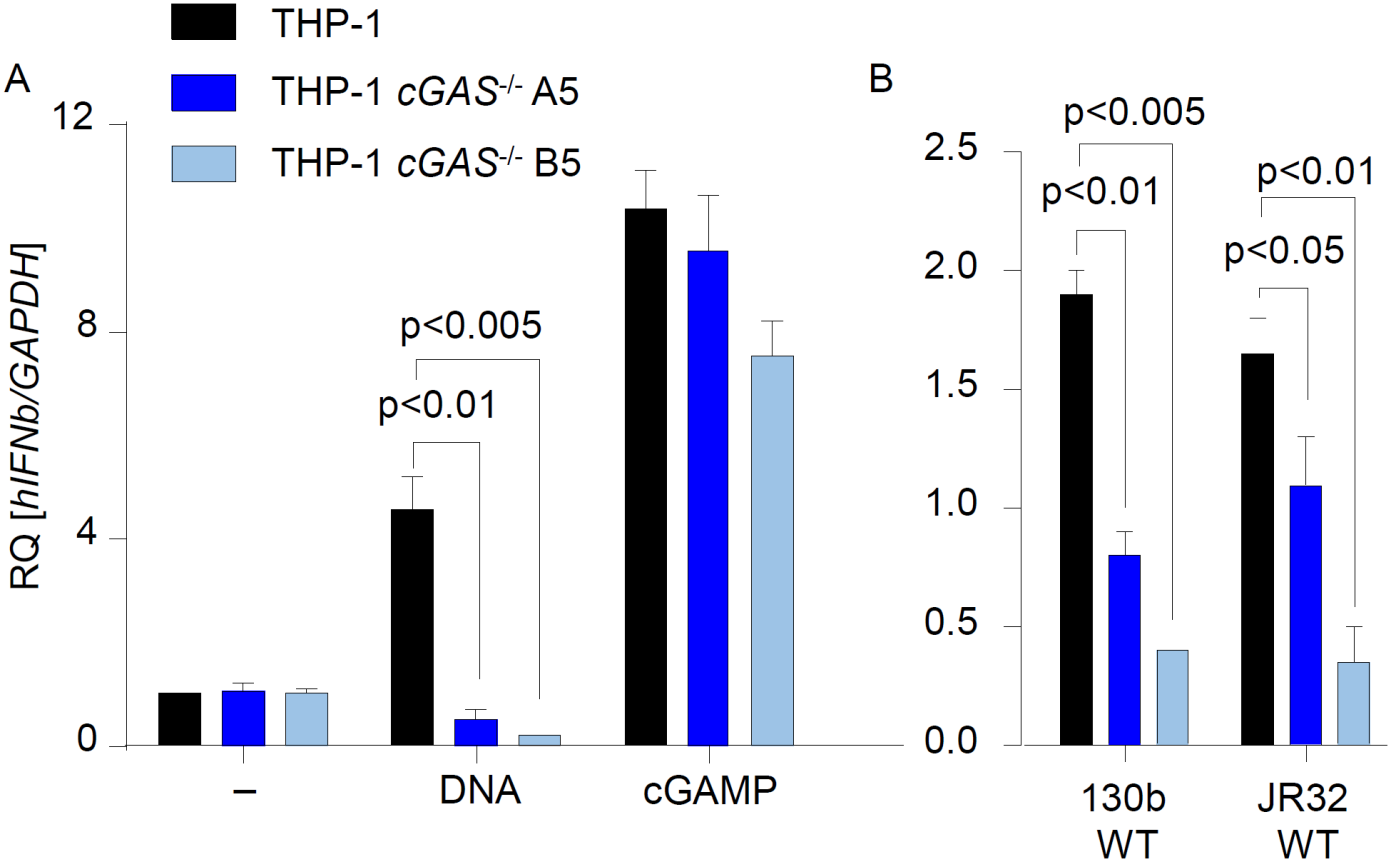
*L*. *pneumophila* infection and stimulation with DNA or cGAMP induce weak cGAS-dependent type I IFN responses in THP-1 cells. WT THP-1 or cGAS-/- THP-1 clones A5 and B5 were allowed differentiation prior to stimulation with either cGAMP or synthetic DNA (A) or infection with two different strains of *L*. *pneumophila* (B). *IFNB* expression was determined by qRT-PCR. Data represent mean ± SEM of 2 independent experiments carried out in duplicates. Analyses were performed by employing the Mann-Whitney U Test. Comparisons with a p < 0.05 were considered significant.

Homozygous HAQ PBMCs were strongly impaired but not blunted in inducing type I IFN responses to *L*. *pneumophila* infection or stimulation with DNA or cGAMP (Fig 3A, S5A Fig), suggesting that HAQ STING possesses largely reduced but not blunted activity. In line with this suggestion, THP-1 cells, which have previously been shown to express HAQ STING [30,32] and which we confirmed to carry the HAQ *TMEM173/STING* allele in homozygosity, responded only weakly to DNA, cGAMP and *L*. *pneumophila* stimulation (2-10-fold increase in *Ifnb* expression, see Fig 4). These type I IFN responses in THP-1 cells were considerably lower as compared to PBMCs expressing WT STING (100-1000-fold *Ifnb* induction, see Fig 3A). Interestingly, however, deletion of cGAS expression by CRISPR/Cas9-mediated genome editing [33] further decreased the type I IFN responses in THP-1 cells (Fig 4). Taken together, our data demonstrate that endogenous HAQ STING is a hypomorphic variant that is strongly impaired (but not deficient) in mediating type I IFN and pro-inflammatory cytokine responses to cGAMP, synthetic DNA, bacterial DNA and *Legionella* infection.

### Endogenous R232H STING is partly defective in sensing bacterial CDNs but fully functional in mediating responses to DNA, cGAMP and *L*. *pneumophila* infection

**Fig 5:**
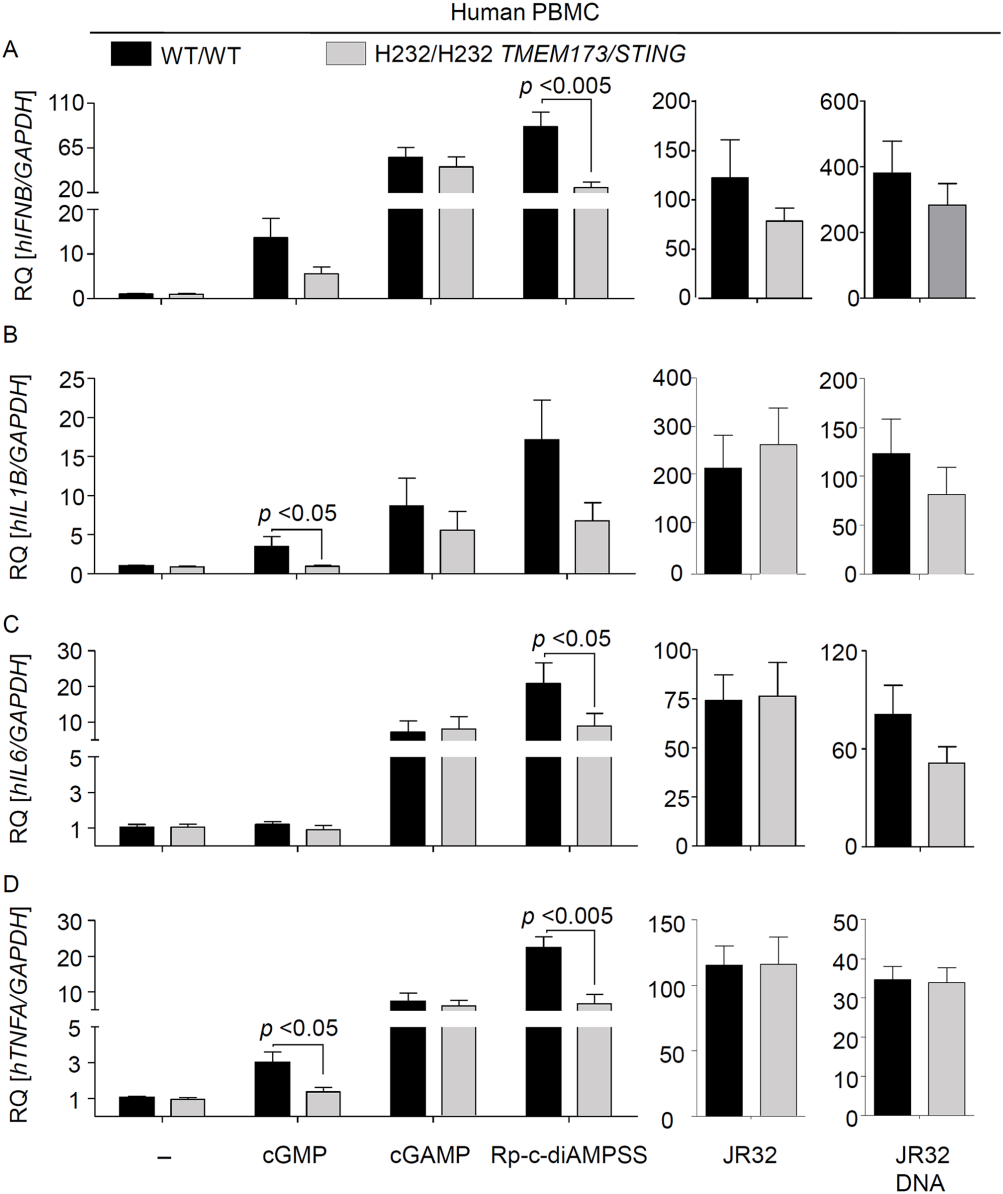
Endogenous R232H STING is partly deficient in sensing bacterial CDN but responds normally to *Legionella* infection or stimulation with DNA. (A-D) PBMCs from healthy volunteers (N = 3 for WT and N = 3 for R232H) were isolated by density gradient centrifugation. 7 d after isolation cells were infected for 6 h with *L*. *pneumophila* at MOI 10 or stimulated for the same period with 1 ug/ml 2´-3´cGAMP, Rp-c-diAMPSS, cGMP or either bacterial DNA at a concentration of 1 ug/ml. RNA was isolated and the expression of *IFNB* (A), *IL1B* (B), and *IL6* (C) and *TNFA* (D) was determined by qRT-PCR. Data are shown as the RQ of specified mRNAs. Data represent the mean ± SEM of 3 independent experiments carried out in triplicates. Differences were assessed with the Mann-Whitney U Test. Comparisons with a p < 0.05 were considered significant.

HEK293 cells expressing a mutated murine STING with an alanine instead of arginine 231 (R231A) respond normally to DNA but not to bacterial CDNs [20]. The corresponding human R232H allele is the third most common *TMEM173/STING* allele [29], and has also been shown to be defective in sensing bacterial CDNs when overexpressed [28,30]. In order to examine the function of endogenous R232H STING and the relevance of c-diGMP sensing for host responses to *L*. *pneumophila*, we screened healthy volunteers for carriage of this allele, isolated cells from 3 individuals harboring the R232H allele in homozygosity, and compared them with cells expressing WT STING. In agreement with previous studies, we found that human cells expressing R232H STING were partly impaired in sensing cGMP and Rp,Rp-c-diAMPSS (a Rp,Rp-isomer of the di-thiophosphate analogue of the bacterial second messenger c-diAMP) (Fig 5A–5D, S8 Fig). In contrast, expression of the R232H allele did not affect type I IFN or pro-inflammatory cytokine responses to *L*. *pneumophila* infection or DNA or cGAMP stimulation. Moreover, the R232H SNP did not affect replication of *L*. *pneumophila* in human cells (S9 Fig). These data indicate that endogenous human R232H STING is partly defective in sensing bacterial CDNs and that recognition of c-diGMP is not critically involved in human cell interactions with *L*. *pneumophila*.

### cGAS and STING contribute to anti-bacterial host defense against *Legionella* infection in mice

**Fig 6:**
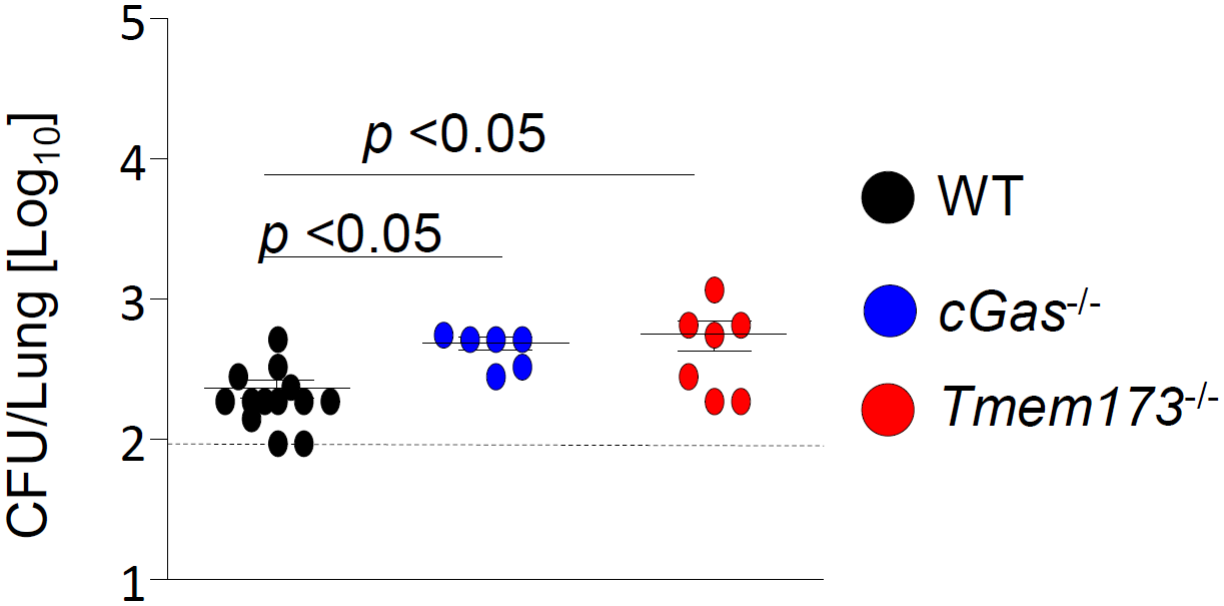
STING contributes to the antibacterial defense in mice infected with *L*. *pneumophila*. WT, cGAS- and STING-deficient mice were intranasally infected with 1×10^6^ *L*. *pneumophila* WT and the bacterial loads in the lungs were assessed 144 h p.i. Data represent mean ± SEM of 6–13 mice per group. Comparisons were performed with the Mann-Whitney U Test. Comparisons with p < 0.05 were considered significant.

Next, we investigated the relevance of the cGAS/STING-dependent pathway for antibacterial defense *in vivo*. cGAS- and STING-deficient mice as well as WT controls were intranasally infected with *L*. *pneumophila*. 6 days after infection, we observed enhanced (2–3 fold) bacterial loads in the lungs of *cGas*^*-/-*^ and *Tmem173*^-/-^ mice as compared to WT controls (Fig 6), demonstrating that the cGAS/STING pathway contributes to antibacterial defense against *L*. *pneumophila in vivo*.

### Carriage of HAQ *TMEM173/STING* might predispose individuals to infection

Finally, we tested for a potential association between HAQ and R232H *TMEM173/STING* carriages and susceptibility towards *L*. *pneumophila* infection. In an exploratory analysis, allele frequencies and genotypes were compared between 59 Legionnaires´ disease patients and 100 healthy controls of similar age and sex distribution. The frequency of HAQ *TMEM173/STING* (but not R232H *TMEM173/STING*) was significantly increased among cases (0.18) as compared to controls (0.075) (Table 1); an unadjusted analysis showed that carriage of the haplotype almost tripled the odds of being a legionellosis patient in this cohort (p = 0.028; OR 2.69; 95%CI, 1.16–6.27). The HAQ haplotype remained associated with the disease when the analysis was performed with an adjustment for age and gender (p = 0.01; OR 2.70, 95% CI 1.24–5.86; logistic regression with dominant genetic model).

**Table 1:** Distribution of *TMEM173/STING* HAQ and R232H in German patients and healthy controls.

	Controls	Cases	*p*	OR (95% CI)
N	100	59		
Proportion female %(n)	39.0 (39)	35.6 (21)	0.669	
Age median (Q_1_ –Q_3_)	71.5 (62.75–80)	72.0 (62.5–81.5)	0.551	
*TMEM173/STING* HAQ Freq.*	0.075	0.178		
Heterozygous %(n)	15.0 (15)	28.8 (17)	0.01	2.70 (1.24–5.86)^†^
Homozygous %(n)	0	3.4 (2)		
*TMEM173/STING* R232H Freq.*	0.15	0.11		
Heterozygous % (n)	28.0 (28)	22.0 (13)	0.336	0.69 (0.32–1.46) ^†^
Homozygous % (n)	1.0 (1)	0		

*, HAQ or R232H frequencies = number of individuals with HAQ haplotype/total number of individuals

^†^, dominant genetic model adjusted for gender and age

To validate these findings, we examined another case control cohort (N = 91 Legionnaires´ disease patients and 88 controls) from a flower show outbreak in the Netherlands in 1999 that has been described in detail previously (S1 Table) [34–37]. The HAQ haplotype was present in 23 cases and 12 controls and associated with increased susceptibility to Legionnaires´ disease in an unadjusted analysis (OR 2.24, 95% CI 1.03–5.31; logistic regression with dominant genetic model). In an analysis adjusted for age and gender, the HAQ haplotype remained associated with Legionnaires´ disease (p = 0.013; OR 2.29, 95% CI 1.04–5.24) (Table 2). In contrast, we did not find a significant association between R232H carriage and susceptibility to infection, although there was a trend towards increased R232H frequency in the Dutch patient cohort that we did not see in the German patients. Together, these data provide evidence in two separate European populations that the HAQ haplotype is associated with increased susceptibility to Legionnaires’ disease.

**Table 2:** Distribution of *TMEM173/STING* HAQ and R232H in patients and healthy controls from Netherlands cohort.

	Control	Cases	*p*	OR (95% CI)
N	88	91		
Proportion female, %(n)	50 (44)	38,4 (35)	0.128	
Age median (Q_1_ –Q_3_)	49.6 (35.2–56.1)	64.7 (54.2–71.5)	< 0.001	
*TMEM173/STING* HAQ Freq.*	0.136 (12/88)	0.252 (23/91)		
Heterozygous % (n)	11.4 (10)	24.2 (22)	0.013	2.29 (1.04–5.24)†
Homozygous % (n)	2.3 (2)	1.1 (1)		
*TMEM173/STING* R232H Freq.*	9.2 (16/174).	16.1 (30/185)		
Heterozygous % (n)	13.8 (12/87)	16.1 (14/93)	0.09	1.85 (0.9–3.80)
Homozygous % (n)	2.3 (2/87)	9.2 (8/93)		

*, HAQ and R232H frequencies = number of individuals with HAQ haplotype/total number of individuals

^†^, dominant genetic model adjusted for gender and age

## Discussion

Genetic variations in different Toll-like receptors (TLRs) and downstream signaling molecules are known to affect innate immune sensing and susceptibility of human diseases [38–40]. Polymorphisms in the genes encoding TLR4, -5 and -6, for example, have been associated with increased risk of Legionnaires’ disease [37,41]. Previous studies also revealed considerable heterogeneity of human *TMEM173/STING* [27,28]. The rare gain-of-function alleles A154S, V155M, V147L are associated with elevated type I IFN production and vasculopathy [42]. In contrast, the common HAQ variant was found to induce less basal type I IFN in the absence of exogenous stimuli as compared to wild-type STING [27,28], and reduced activity in the presence of CDNs [28,29]. Moreover, the R232H isoform of STING was shown to be defective in sensing bacterial CDNs but not cGAMP or DNA [28,30]. The relevance of endogenously expressed HAQ and R232H STING on the sensing of microbes or DNA, as well as a potential linkage between HAQ *TMEM173/STING* and acute infections, however, had not been addressed before.

Here we demonstrate that endogenous HAQ STING is impaired in mediating type I IFN and pro-inflammatory cytokine production in response to *Legionella* infection, bacterial and synthetic DNA, as well as cGAMP. The fact that PBMCs and THP-1 cells expressing HAQ STING in homozygosity are still able to produce some IFN, and that deletion of cGAS in THP-1 cells further reduces these responses, however, indicates that HAQ STING is a hypomorphic rather than a loss-of-function variant.

Importantly, our analyses of two independent cohorts of patients and healthy controls indicated an association between carriage of HAQ *TMEM173/STING* and Legionnaires’ disease. This is the first time that a linkage between HAQ *TMEM173/STING* and susceptibility towards infectious diseases is reported. Our findings indicate that carriage of HAQ *TMEM173/STING* represents a risk factor for Legionnaires’ disease. Moreover, considering that STING is involved in the defense against various pathogens like e.g. *Mycobacterium tuberculosis* and HIV, but weakens immunity against *Plasmodium falciparum* infections [43–45], one could speculate that HAQ *TMEM173/STING* carriage might predispose individuals towards several bacterial and viral infections, while at the same time potentially conferring protection against Malaria. Moreover, since STING also plays an important role in the pathogenesis of DNA-/IFN-driven autoimmune diseases [42], carriers of HAQ *TMEM173/STING* might be protected against these conditions.

Similar to most Gram-negative bacteria, *L*. *pneumophila* produces the second messenger c-diGMP [46,47], and previous studies suggested that sensing of *Legionella* c-diGMP might also play a role in inducing cytokine responses [17]. STING has been identified as a direct sensor of bacterial CDNs [20]. Previous overexpression studies indicated that the third most common *TMEM173/STING* allele R232H encodes for a protein with attenuated ability to recognize bacterial CDNs [28,30]. In agreement to these studies, we found that homozygous carriage of *TMEM173/STING* R232H in primary human cells impaired their ability to sense a bacterial CDN. However we did not observe a reduced cytokine production of R232H cells following infection with *L*. *pneumophila*, suggesting that sensing of *Legionella* CDNs is not required for the innate immune response to this infection. Moreover, we did not find a significant association between carriage of R232H *TMEM173/STING* and Legionnaires’ disease, although the R232H frequency was enhanced by trend in the Dutch patient cohort as compared to the controls (whereas the opposite trend was observed in the German cohort).

We and others recently showed that type I IFNs activate a macrophage-intrinsic resistance pathway that restricts *L*. *pneumophila* [4,8,12,48]. Surprisingly, we observed no difference in *Legionella* replication in macrophages from STING-deficient mice or HAQ carriers as compared to control cells. We do not have a definite explanation for this unexpected result, but speculate that small amounts of STING-independently produced type I IFN [49] might be sufficient to control the infection *in vitro*.

While the cGAS-STING pathway has been primarily associated with type I IFN responses to microbial infections, our results illustrate that sensing of *L*. *pneumophila* by cGAS/STING does not only stimulate type I IFN responses, but also significantly contributes to the production of other pro-inflammatory cytokines such as IL-1β and IL-6 or TNFα. This observation is in line with previous reports demonstrating STING-dependent NF-κB activation [50]. Considering that both IFNs as well as pro-inflammatory cytokines are known to be required for controlling *L*. *pneumophila* infection in the lung [4–8], a reduced production of these mediators might explain why lack of STING or cGAS in mice or expression of HAQ STING in humans enhances the susceptibility towards *L*. *pneumophila* infection. The reason for the rather small (but significant) effect of cGAS or STING deficiency on bacterial burden in our mouse model might be that mice are (probably due to an apparently more effective NAIP(5)/NLRC4 inflammasome) generally more resistant to *L*. *pneumophila* infection that humans.

In summary, we show that cGAS/STING contributes to the antibacterial defense against *L*. *pneumophila* infection, reveal that the hypomorphic STING variant HAQ negatively affects the antibacterial immune response, and indicate that HAQ *TMEM173/STING* carriage predisposes to Legionnaires ‘disease.

## Materials and methods

### Ethics statement

For healthy volunteers from whom PBMCs were isolated, written informed consent was obtained and the study procedures were approved by the local ethics committee (Charité-Universitätsmedizin Berlin). Samples from the German Legionnaires’ disease patients were provided by the CAPNETZ foundation. This prospective multicenter study (German Clinical Trials Register: DRKS00005274) was approved by the ethical review board of each participating clinical center (Reference number of leading Ethics Committee “Medical Faculty of Otto-von-Guericke-University in Magdeburg”: 104/01 and “Medical School Hannover”: 301/2008) and was performed in accordance with the Declaration of Helsinki. All patients provided written informed consent prior to enrolment in the study. With regard to the Dutch case control study, approval for was obtained from the human subjects’ review boards at the University of Amsterdam Medical Center and the University of Washington Medical Center (IRB protocol 1356). All participants gave written informed consent. All animal experiments were carried out in strict adherence to the German law (Tierschutzgesetz, TierSchG), following the approval of the corresponding institutional (Charité-Universitätsmedizin Berlin) and governmental animal welfare authorities (LAGeSo Berlin, approval ID G0440/12).

### Bacterial strains

The *L*. *pneumophila* serogroup 1 strains JR32 and 130b as well as the isogenic mutant strains *ΔdotA* and *ΔsdhA* have been described previously [12,51,52]. Bacterial DNA was purified using the QIAamp DNA Mini Kit (Qiagen, Hilden, Germany).

### Murine model of Legionnaires’ Disease

Anesthetized 8–16 weeks old, female WT, *cGas*^*-/-*^ and *Tmem173*^*-/-*^ mice on C57BL/6 background [53] were intranasally infected with 1×10^6^ *L*. *pneumophila* JR32, and bacterial numbers in the lungs were counted as previously described [4,12].

### Cell transfection and infection

Mouse BMDMs were infected with the aforementioned strains of *L*. *pneumophila* at MOI 10, centrifuged at 200 *g* for 5 min and then incubated for 6 h for qPCR analysis or for 16–18 h for ELISA at 37°C. Bacterial or synthetic nucleic acids were transfected into the cells using Lipofectamine 2000 (Life Technologies, Darmstadt, Germany) at a concentration of 1 ug/ml. 2’3’-cGAMP was added into the cell media without any transfection reagent at a concentration of 5 ug/ml. Where indicated, BMDMs were transfected 48 h prior to infection with control non-silencing siRNA or with a specific siRNA targeting cGAS using HiPerfect (Qiagen, Hilden, Germany).

### Subjects

Human peripheral blood was collected from healthy adult volunteers expressing HAQ/HAQ (N = 4), R232H/R232H (N = 3), WT/HAQ (N = 7) or WT/WT (N = 10) *TMEM173/STING* and belonging to a cohort group (N = 564) collected at the Institute of Microbiology, Charité-Universitätsmedizin Berlin. The association study between the HAQ and R232H haplotypes and Legionnaires’ disease was carried out first from DNA samples obtained from 59 German adult patients with confirmed *L*. *pneumonia*-induced community-acquired pneumonia (CAP). Samples were provided by the CAPNETZ competence network, a German multi-center prospective cohort study for CAP [54]. The control groups consisted of a subgroup of healthy adults of similar age and sex distribution (N = 100) from the PolSenior program, an interdisciplinary project, designed to evaluate health and socio-economic status of the Polish Caucasians aged ≥65 y [55]. Enrollment of the cases and controls from the Legionnaires’ disease outbreak in the Netherlands has been described previously [34–37]. Of the 188 cases (all adults) identified in the original investigation of the outbreak, 141 consented for the study. 18 individuals died and no DNA was available for genotyping. 95 cases were available with both DNA and epidemiologic data for STING genotyping. Controls (N = 95, all adults) were drawn from the exhibitioners who worked at the flower show and were at high risk for exposure to *L*. *pneumophila*. Genomic DNA was purified form peripheral blood leukocytes from 10 ml of blood.

### *TMEM173/STING* genotyping

Genomic DNA from the volunteers belonging to the cohort group collected at the Institute of Microbiology, Charité-Universitätsmedizin Berlin was extracted from buccal mucosa using the Gentra Puregene Buccal Cell Kit from Qiagen, according to the manufacturer’s instructions. DNA from cases and controls from the CAPNETZ competence network and the PolSenior program respectively, were isolated from whole blood. Genotyping of *TMEM173/STING* R71H (rs11554776), G230A (rs78233829) and R293Q (rs7380824) in the above mentioned samples was carried out by PCR employing fluorescence-labeled hybridization FRET probes followed by melting curve analysis in a LightCyler 480 (Roche Diagnostics). Primer and probes used were as follows: rs11554776: f-primer: ggagtgacacacgttgg, r-primer: gcctagctgaggagctg, simple probe: LC640-ctggagtggaXItgtggcgcag-PH; rs78233829: f-primer: gggtctcactcctgaatcaggt, r-primer: ccgatccttgatgcaagca, anchor probe: LC640-cagtttatccaggaagcgaatgttggg-PH, sensor probe: ggtcagcggtctgctgg-FL; rs7380824: f-primer: accctggtaggcaatga, r-primer: gcttagtctggtcttcctcttac, anchor probe: LC640-ggcctgctcaagcctatcctcccgg-PH, sensor probe: cctcaagtgtccggcagaagagtt-FL; rs1131769: f-primer: cccactcccctgcacactt, r-primer: tggataaactgcccaagcagac, anchor probe: LC640-aggatcgggtttacagcaacagca-PH, sensor probe: ggtgaccatgctggcatc-FL.

Genomic DNA from cases and controls from the Legionnaires’ disease outbreak in the Netherlands was isolated from whole blood, and genotyping of selected SNPs was performed using a Fluidigm Biomark 96 x 96 chip (Fluidigm, Inc.). Cluster plots were visually inspected to ensure accurate genotyping calls. SNPs were manually assessed for data quality and only high-quality calls were accepted. 91 cases and 88 controls had high-quality genotyping data available for all three SNPs for analysis. Genotypes were assessed for Hardy-Weinberg equilibrium (HWE) with a Chi-square test comparing observed and expected frequencies in the control population. No SNPs violated HWE (P < 0.001).

### Infection and stimulation of human peripheral blood mononuclear cells

50 mL of whole blood were drawn from healthy volunteers and peripheral blood mononuclear cells were isolated by gradient centrifugation using Histopaque-1077 (Sigma-Aldrich, Taufkirchen, Germany). Briefly, whole blood was diluted 1:1 with phosphate buffered saline solution (PBS) without calcium or magnesium and layered onto 20 ml Histopaque-1077. The gradient was centrifuged at 800 x g for 25 min at room temperature, and the PBMC were collected from the interface. PBMC were then washed twice with PBS and resuspended in RPMI medium supplemented with 10% FCS and 1% l-glutamine. Cell media was replaced 24 h after plating and half of the media was further replaced every 2 d and the cells were cultured for 7 d before infection or stimulation. Infection with *L*. *pneumophila* was performed at MOI 10 and 50. Bacterial DNA or synthetic nucleic acids were transfected into the cells using concentrations of 0.2 or 1 ug/ml. 2´3-cGAMP and RpRp-c-diAMPSS were added into the cell media at concentrations of 1 or 5 μg/ml, and R878 was used at a concentration of 1 μg/ml. All procedures used for PBMC infection or stimulation were performed as described before for mouse BMDMs.

### Human monocytic THP-1 cells

cGAS-deficient [33] and control THP-1 cells were maintained under normal culture conditions. For induction of cell differentiation into a macrophage-like state, cells were re-suspended in culture medium containing 80 nM phorbol myristate acetate (PMA) for 48 h.

### qRT-PCR

Total RNA was isolated from cultured cells or lung homogenates using the PerfectPure RNA purification system (5 Prime) or Trizol (Life Technologies, Darmstadt, Germany), respectively. Total RNA was reverse-transcribed using the high capacity reverse transcription kit (Applied Biosystems, Darmstadt, Germany), and quantitative PCR was performed using TaqMan assays (Life Technologies, Darmstadt, Germany) or self-designed primer sets, on an ABI 7300 instrument (Applied Biosystems, Darmstadt, Germany). The input was normalized to the average expression of GAPDH and relative expression (relative quantity, RQ) of the respective gene in untreated cells or PBS-treated mice was set as 1.

### ELISA

Concentrations of IL-1β, IL-6, TNFα and IFNγ were quantified by commercially available sandwich ELISA kits (eBioscience, Frankfurt, Germany) as well as human and mouse IP-10 (Life Sciences, Darmstadt, Germany). Protein concentrations were determined in a FilterMax F5 Multi-Mode Microplate Reader (Molecular devices, Sunnyvale, CA, USA) at 450 nm.

### Bacterial replication analysis

Murine BMMs and PBMCs were infected with *L*. *pneumophila* at MOI 0.1 and intracellular bacterial replication was estimated with a CFU assay. Briefly, 30 min after infection cells were washed with PBS and cell media containing 50 ug/ml gentamycin was supplemented. After 1 h, cells were washed once more and fresh medium was added. Cell lysis was performed by adding 1% saponin 1, 24, 48 and 72 h.p.i and CFUs were estimated by plating different serial dilutions of the resultant cell suspension in buffered charcoal yeast extract (BCYE) agar.

### Statistics and genetic analysis

Data analysis was performed using the Prism software (GraphPad Software, La Jolla, CA). Groups were compared using a two-tailed Mann-Whitney U test. The association analysis in the German cohorts was performed through the Chi-Square test for association and calculation of odds ratio using a dominant genetic model (comparing WT individuals (no HAQ haplotype) to those who had 1 or 2 copies of HAQ or R232H). Odds ratios were adjusted for age and gender through a logistic regression analysis using SPSS (IBM Corporation, Armonk, NY). A Fisher´s exact test together with calculation of exact confidence intervals were used if applicable. Differences with p<0.05 were regarded as significant. Similarly, the association analysis in the Dutch cohorts was performed with a dominant genetic model using Stata 13 (Stata Corp, College Station, TX) and the user-written package “genass” [56]. The presence of the HAQ or R232H haplotypes was defined as an individual who was heterozygous or homozygous for any of the two STING variants.

## Supporting information

S1 Fig Replication of *L*. *pneumophila* in murine WT and *Tmem173-/-* macrophages and IP-10 production. (A) WT and *Tmem173-/-* mouse BMDMs were infected with *L*. *pneumophila* at MOI 0.1, and bacterial loads were analyzed at the indicated time points. Data represent mean ± SEM of 5 independent experiments carried out in triplicates. (B) WT and STING-deficient BMDMs were infected with *L*. *pneumophila* JR32 for 16–18 h, and production of IP-10 was measured by ELISA. Data represent mean ± SEM of 4 independent experiments carried out in duplicates. Comparisons with a *p* < 0.05 were considered significant.
(PDF)

S2 Fig siRNA-mediated inhibition of cGAS reduces type I IFN responses against *L*. *pneumophila* in murine macrophages. BMDMs were transfected with a control siRNA or a siRNA sequence targeting *cGas* 48 h prior to infection; the expression of (A) *cGas*, (B) *Ifnb* and (C) *Irg1* was quantified by qRT-PCR and the input normalized to the average expression of *Gapdh* and the relative expression of the respective gene in untreated cells. Data are shown as mean + SEM of three independent experiments, measured in technical duplicates. Analyses were performed through the Mann-Whitney U Test. Comparisons with a *p* < 0.05 were considered significant.
(PDF)

S3 Fig STING deficiency affects *L*. *pneumophila*-induced production of pro-inflammatory cytokines. (A-C) Cytokine protein production was assessed by sandwich ELISA of supernatants from WT and *Tmeme173-/-* BMDMs infected for 16–18 h with *L*. *pneumophila* JR32 WT. Analyses were performed through the Mann-Whitney U Test. Data represent mean ± SEM of 4 independent experiments carried out in duplicates. Comparisons with a *p* < 0.05 were considered significant.
(PDF)

S4 Fig Replication of *L*. *pneumophila* in human macrophages is not affected by carriage of HAQ *TMEM173/STING*. PBMCs from healthy volunteers (N = 4, per group) were isolated by density gradient centrifugation. 7 d after isolation, cells were infected with *L*. *pneumophila* at MOI 0.1 and bacterial numbers were counted at the indicated time points. Data represent mean ± SEM of 4 independent experiments carried out in triplicates.
(PDF)

S5 Fig Cells from homozygous HAQ *TMEM173/STING* carriers are impaired in cytokine production in response to cGAMP, *L*. *pneumophila*, and bacterial DNA. **(A-D)** PBMCs from healthy volunteers (N = 4 for WT, N = 4 for HAQ) carrying the WT variant of the *TMEM173/STING* gene or the HAQ allele in homozygosity were isolated as described above and infected for 16 to 18 h with *L*. *pneumophila* at MOI 50 or stimulated for the same period with 5 ug/ml 2´-3´cGAMP or of 1 ug/ml bacterial DNA. Protein production of (A) IP-10, (B) IL-1β, (C) IL-6 and (D) TNFα, was assessed by sandwich ELISA of cell supernatants. Data are shown as mean + SEM of four independent experiments, measured in technical triplicates. Analyses were performed through the Mann-Whitney U Test. Comparisons with a *p* < 0.05 were considered significant. # Not detectable.
(PDF)

S6 Fig Cells from homozygous HAQ *TMEM173/STING* carriers are competent in responding to TLR7/8 activation. PBMCs from healthy volunteers (N = 3 for WT, N = 3 for HAQ) were isolated by density gradient centrifugation. 7 d after isolation cells were stimulated for 6 h with 1 ug/ml R848. RNA was isolated and the expression of *IFNB* and *IL1B* was determined by qRT-PCR. Data are shown as the RQ of specified mRNAs. Data represent the mean ± SEM of 3 independent experiments carried out in duplicates. Differences were assessed with the Mann-Whitney U Test. Comparisons with a p < 0.05 were considered significant.
(PDF)

S7 Fig Cells from heterozygous HAQ *TMEM173/STING* carriers are partially impaired in *IFNB* induction in response to cGAMP and *L*. *pneumophila*. **(A-D)** PBMCs from healthy volunteers (N = 7 for WT/WT, N = 7 for WT/HAQ) carrying the WT variant of the *TMEM173/STING* gene or the HAQ allele in heterozygosity were isolated as described above and infected for 6 h with *L*. *pneumophila* at MOI 10 or 50 or stimulated for the same period with 5 ug/ml 2´-3´cGAMP or of 0.2 or 1 ug/ml bacterial DNA. RNA was isolated and the expression of *IFNB*, *IL1B*, *IL6 and TNFA* was determined by qRT-PCR. Data are shown as the RQ of specified mRNAs. Data represent the mean ± SEM of 7 independent experiments carried out in triplicates. Differences were assessed with the Mann-Whitney U Test. Comparisons with a p < 0.05 were considered significant.
(PDF)

S8 Fig Carriage of the R232H *TMEM173/STING* affects production of pro-inflammatory cytokines following stimulation with bacterial CDN but not in response to *Legionella* infection or stimulation with DNA. (A-D) PBMCs from healthy volunteers (N = 3 for WT, N = 3 for R232H) carrying the WT or the R232H allele in homozygosity were isolated and infected for 16 to 18 h with *L*. *pneumophila* at MOI 50 or stimulated for the same period with 5 ug/ml 2´-3´cGAMP, 1 ug/ml Rp,Rp-c-diAMPSS or 1 ug/ml bacterial DNA. Production of (A) IP-10, (B) IL-1β, (C) IL-6 and (D) TNFα was assessed by sandwich ELISA of cell supernatants. Data are shown as mean + SEM of three independent experiments, carried out in triplicates. Analyses were performed through the Mann-Whitney U Test. Comparisons with a *p* < 0.05 were considered significant. # Not detectable.
(PDF)

S9 Fig Carriage of the R232H *TMEM173/STING* allele does not affect replication of *L*. *pneumophila* in human cells. PBMCs from healthy volunteers (N = 3 for WT, N = 3 for R232H) were isolated by density gradient centrifugation. 7 d after isolation, cells were infected with *L*. *pneumophila* at MOI 0.1 and bacterial numbers were counted at the indicated time points. Data represent mean ± SEM of 3 independent experiments carried out in triplicates.
(PDF)

S1 Table Demographics of the Netherlands cohort. (DOCX)
