# Supplementary figures and images for: The common HAQ STING variant impairs cGAS-dependent antibacterial responses and is associated with susceptibility to Legionnaires’ disease in humans
Source: PLoS Pathog. 2018 Jan 3;14(1):e1006829. doi: 10.1371/journal.ppat.1006829 (PMC5770077; doi:10.1371/journal.ppat.1006829)

S1 Fig.

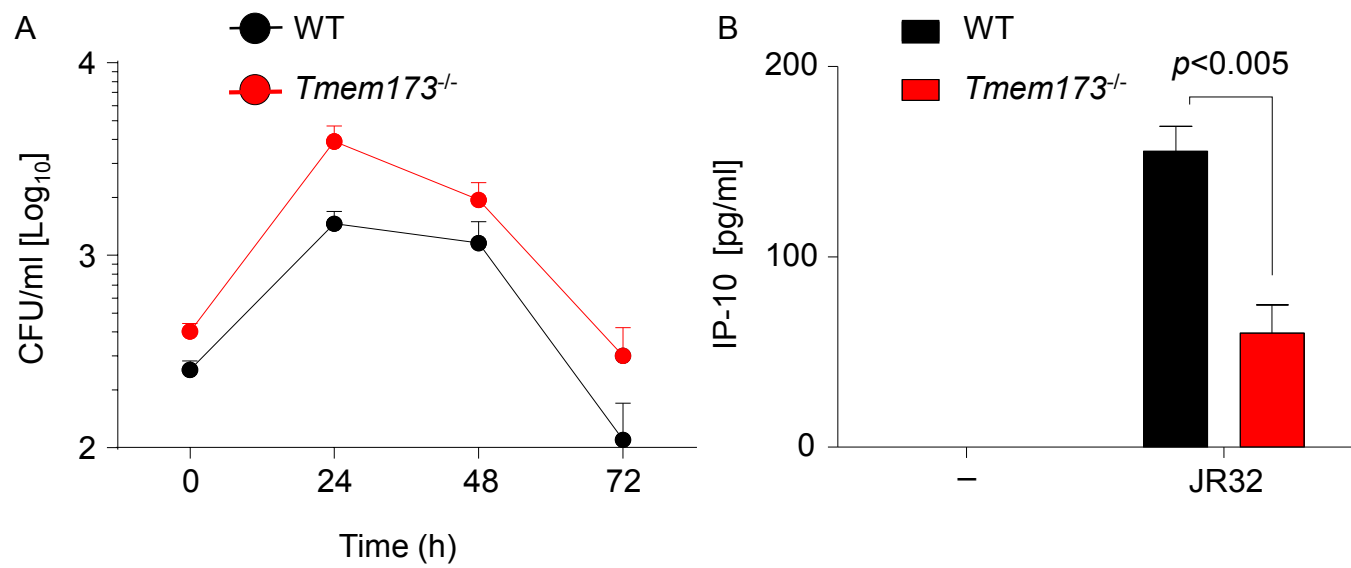

Supplement: S1 Fig — (A) WT and Tmem173-/- mouse BMDMs were infected with L. pneumophila at MOI 0.1, and bacterial loads were analyzed at the indicated time points. Data represent mean ± SEM of 5 independent experiments carried out in triplicates. (B) WT and STING-deficient BMDMs were infected with L. pneumophila JR32 for 16–18 h, and production of IP-10 was measured by ELISA. Data represent mean ± SEM of 4 independent experiments carried out in duplicates. Comparisons with a p < 0.05 were considered significant. (PDF) [file ppat.1006829.s001.pdf]

S2 Fig.

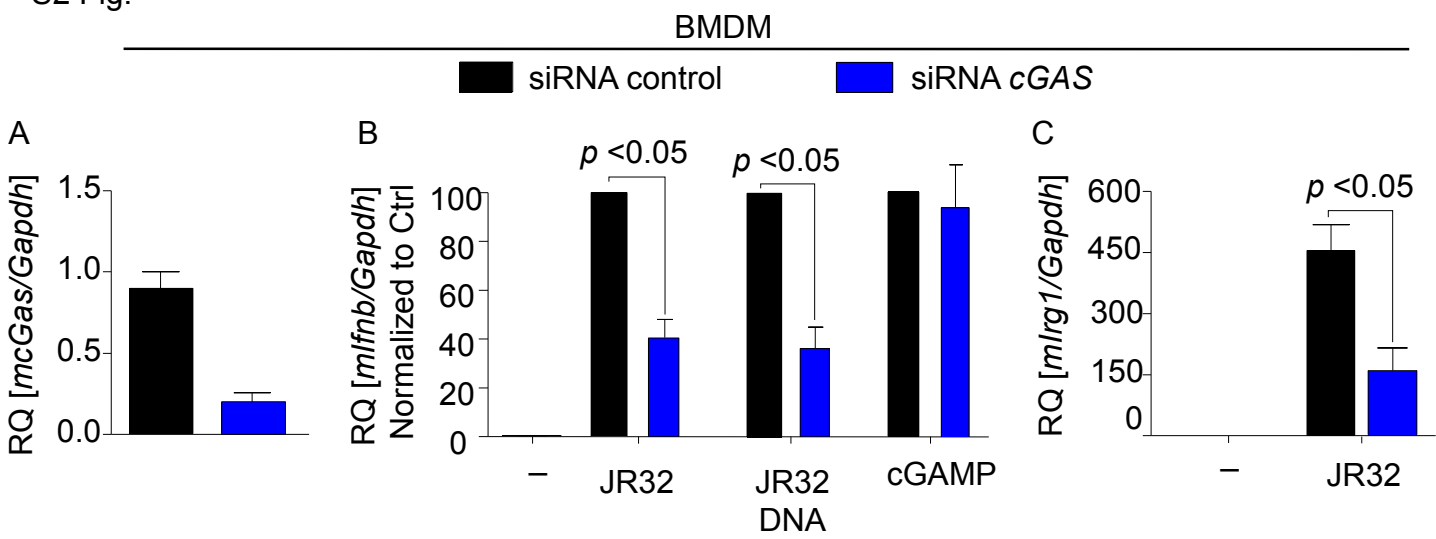

Supplement: S2 Fig — BMDMs were transfected with a control siRNA or a siRNA sequence targeting cGas 48 h prior to infection; the expression of (A) cGas, (B) Ifnb and (C) Irg1 was quantified by qRT-PCR and the input normalized to the average expression of Gapdh and the relative expression of the respective gene in untreated cells. Data are shown as mean + SEM of three independent experiments, measured in technical duplicates. Analyses were performed through the Mann-Whitney U Test. Comparisons with a p < 0.05 were considered significant. (PDF) [file ppat.1006829.s002.pdf]

A

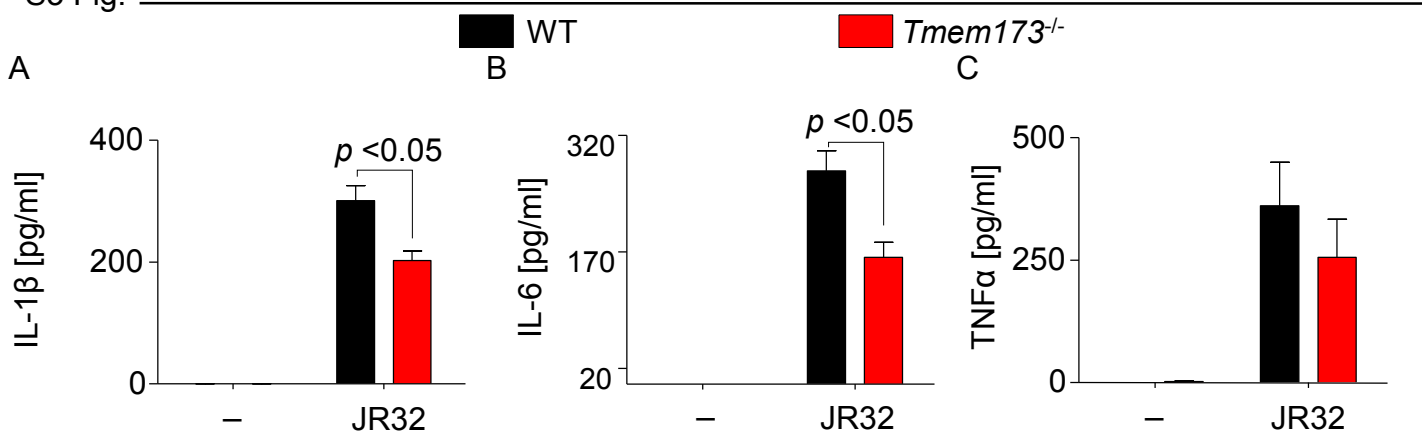

Supplement: S3 Fig — (A-C) Cytokine protein production was assessed by sandwich ELISA of supernatants from WT and Tmeme173-/- BMDMs infected for 16–18 h with L. pneumophila JR32 WT. Analyses were performed through the Mann-Whitney U Test. Data represent mean ± SEM of 4 independent experiments carried out in duplicates. Comparisons with a p < 0.05 were considered significant. (PDF) [file ppat.1006829.s003.pdf]

S4 Fig.

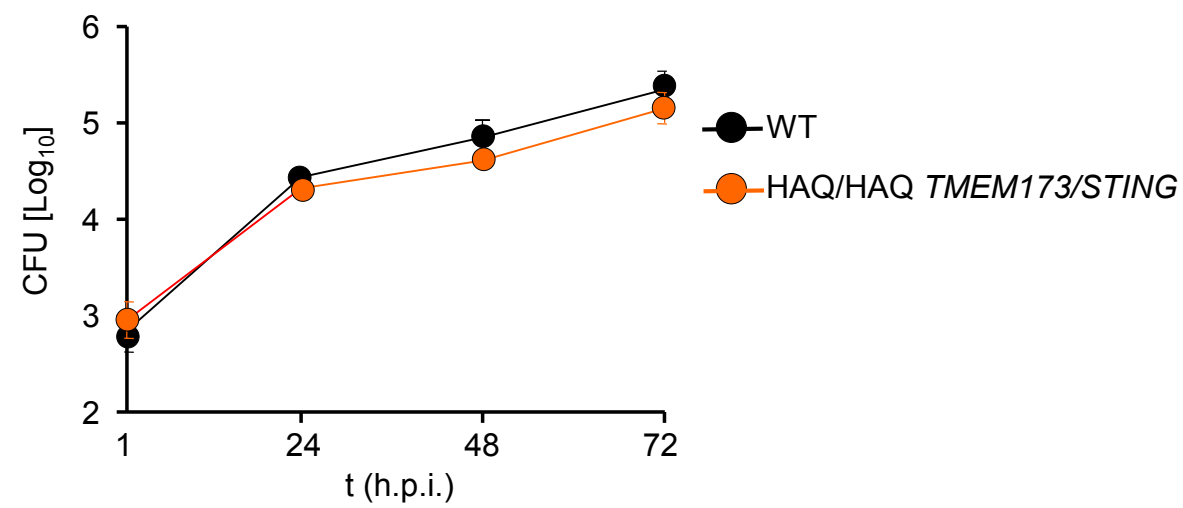

Supplement: S4 Fig — PBMCs from healthy volunteers (N = 4, per group) were isolated by density gradient centrifugation. 7 d after isolation, cells were infected with L. pneumophila at MOI 0.1 and bacterial numbers were counted at the indicated time points. Data represent mean ± SEM of 4 independent experiments carried out in triplicates. (PDF) [file ppat.1006829.s004.pdf]

## Human PBMC

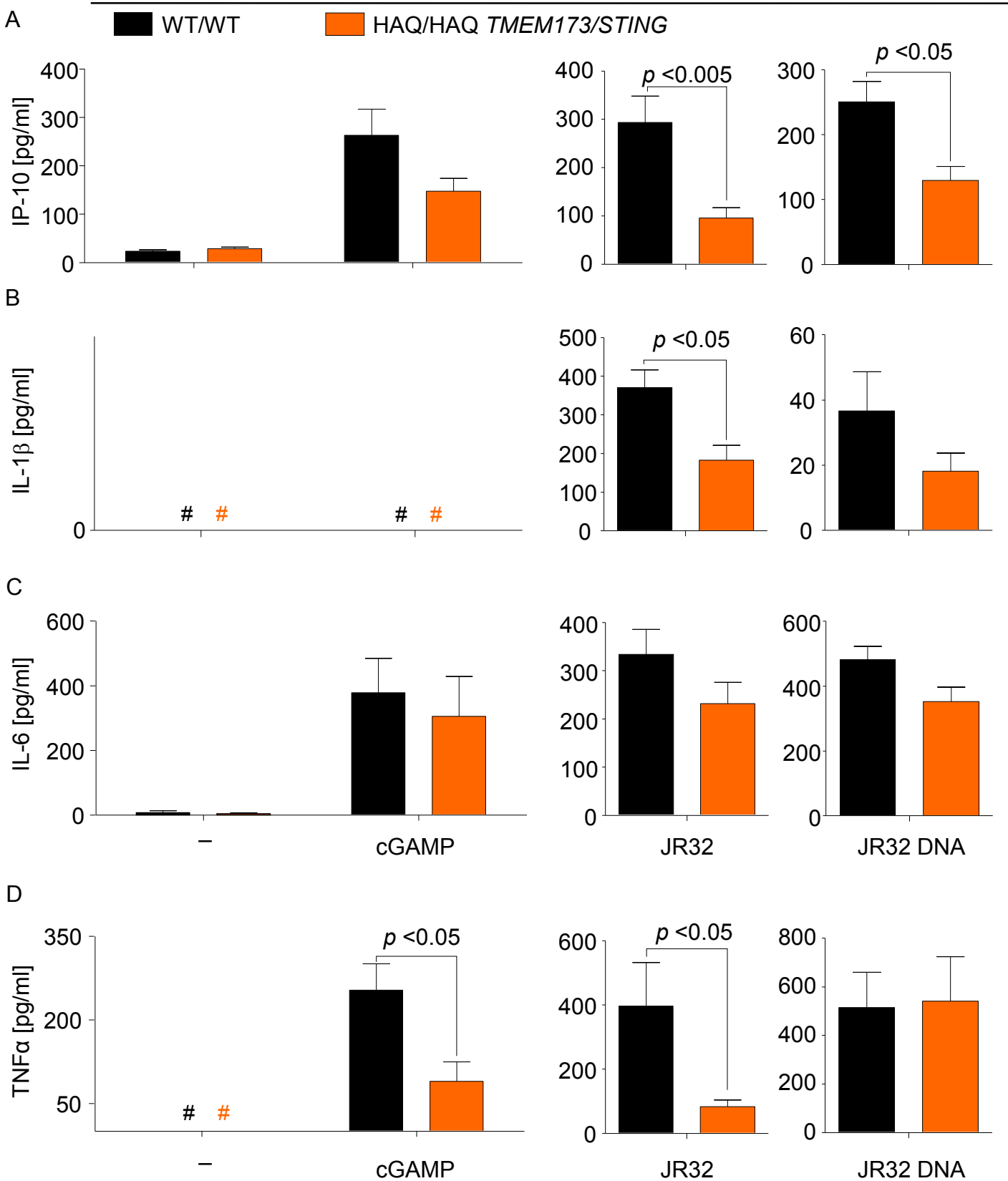

Supplement: S5 Fig — (A-D) PBMCs from healthy volunteers (N = 4 for WT, N = 4 for HAQ) carrying the WT variant of the TMEM173/STING gene or the HAQ allele in homozygosity were isolated as described above and infected for 16 to 18 h with L. pneumophila at MOI 50 or stimulated for the same period with 5 ug/ml 2´-3´cGAMP or of 1 ug/ml bacterial DNA. Protein production of (A) IP-10, (B) IL-1β, (C) IL-6 and (D) TNFα, was assessed by sandwich ELISA of cell supernatants. Data are shown as mean + SEM of four independent experiments, measured in technical triplicates. Analyses were performed through the Mann-Whitney U Test. Comparisons with a p < 0.05 were considered significant. # Not detectable. (PDF) [file ppat.1006829.s005.pdf]

S6 Fig.

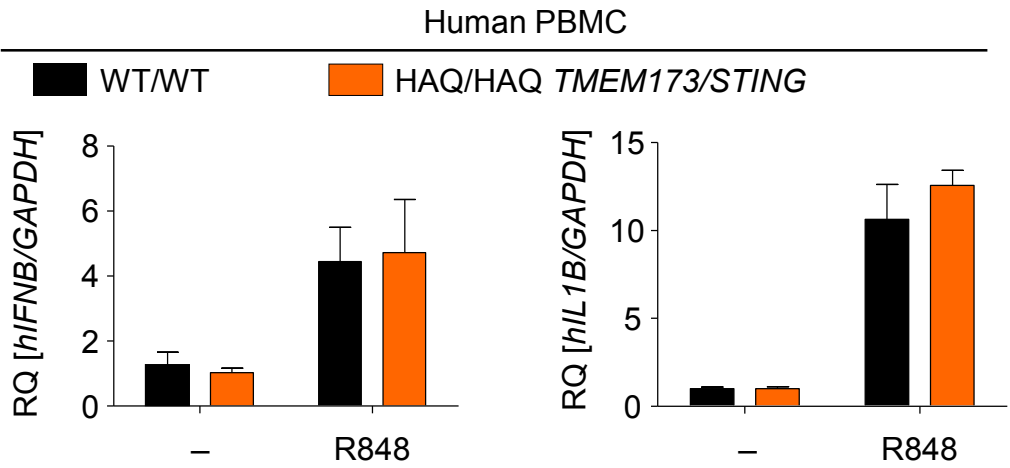

Supplement: S6 Fig — PBMCs from healthy volunteers (N = 3 for WT, N = 3 for HAQ) were isolated by density gradient centrifugation. 7 d after isolation cells were stimulated for 6 h with 1 ug/ml R848. RNA was isolated and the expression of IFNB and IL1B was determined by qRT-PCR. Data are shown as the RQ of specified mRNAs. Data represent the mean ± SEM of 3 independent experiments carried out in duplicates. Differences were assessed with the Mann-Whitney U Test. Comparisons with a p < 0.05 were considered significant. (PDF) [file ppat.1006829.s006.pdf]

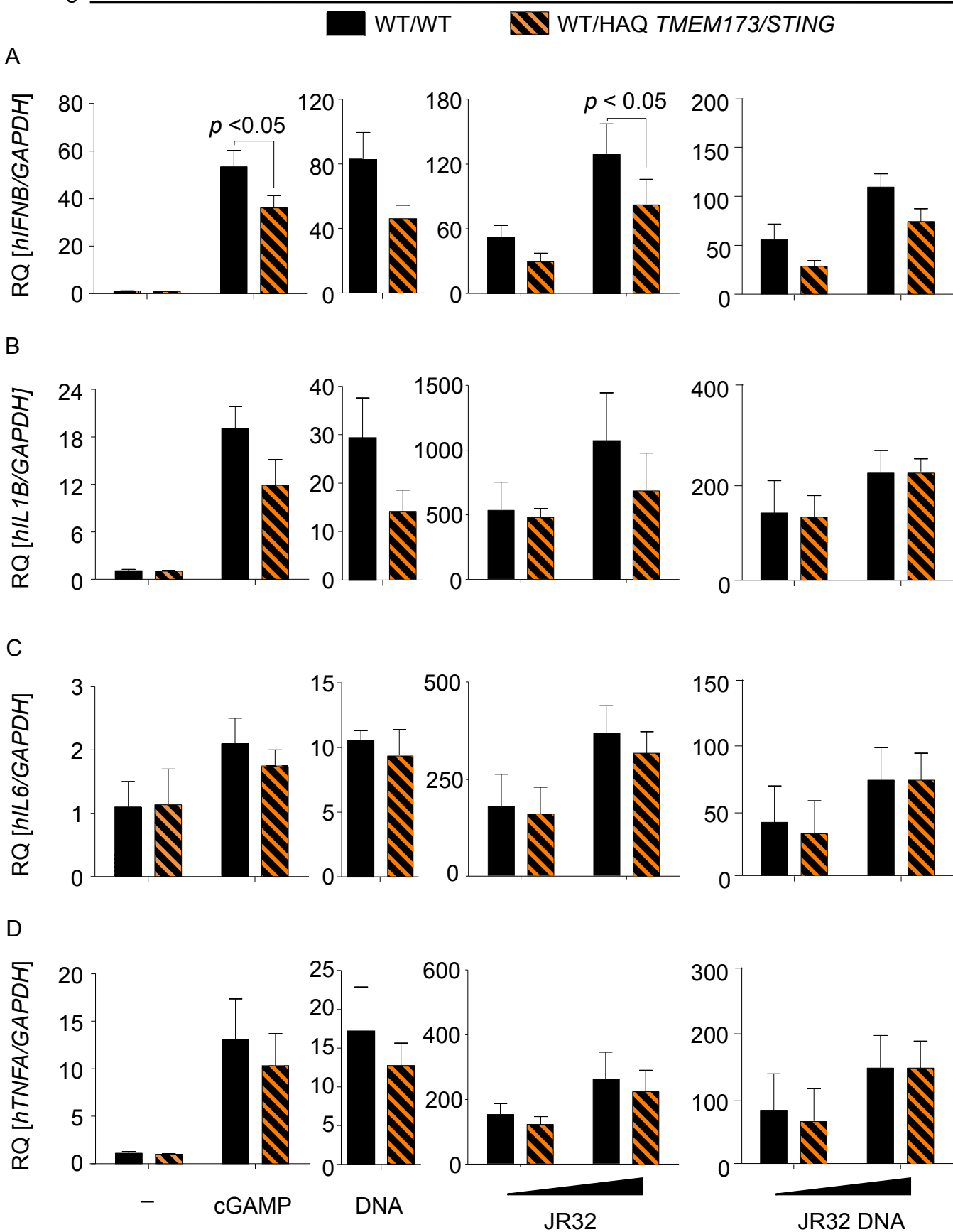

Supplement: S7 Fig — (A-D) PBMCs from healthy volunteers (N = 7 for WT/WT, N = 7 for WT/HAQ) carrying the WT variant of the TMEM173/STING gene or the HAQ allele in heterozygosity were isolated as described above and infected for 6 h with L. pneumophila at MOI 10 or 50 or stimulated for the same period with 5 ug/ml 2´-3´cGAMP or of 0.2 or 1 ug/ml bacterial DNA. RNA was isolated and the expression of IFNB, IL1B, IL6 and TNFA was determined by qRT-PCR. Data are shown as the RQ of specified mRNAs. Data represent the mean ± SEM of 7 independent experiments carried out in triplicates. Differences were assessed with the Mann-Whitney U Test. Comparisons with a p < 0.05 were considered significant. (PDF) [file ppat.1006829.s007.pdf]

S8 Fig.

Human PBMC

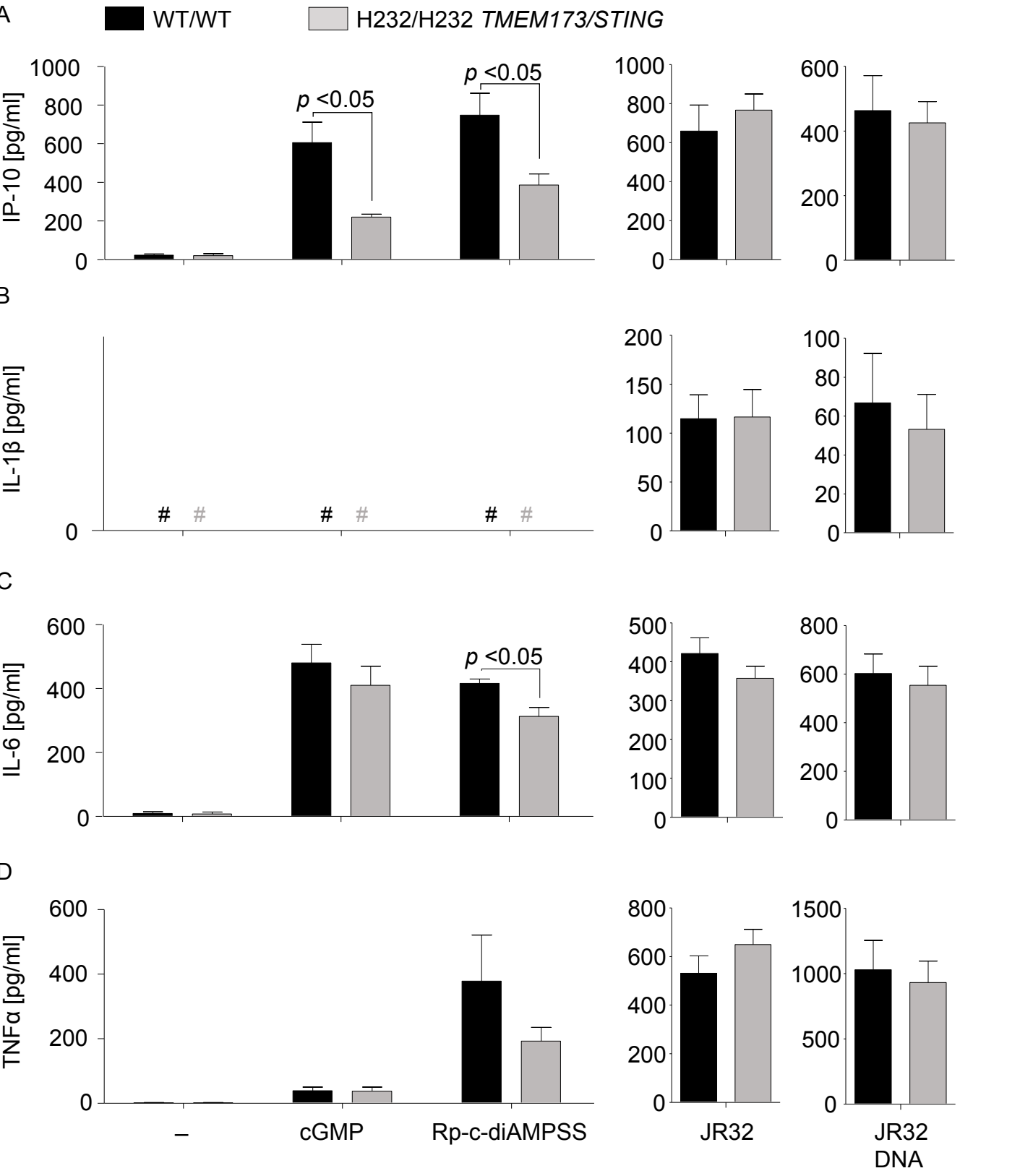

Supplement: S8 Fig — (A-D) PBMCs from healthy volunteers (N = 3 for WT, N = 3 for R232H) carrying the WT or the R232H allele in homozygosity were isolated and infected for 16 to 18 h with L. pneumophila at MOI 50 or stimulated for the same period with 5 ug/ml 2´-3´cGAMP, 1 ug/ml Rp,Rp-c-diAMPSS or 1 ug/ml bacterial DNA. Production of (A) IP-10, (B) IL-1β, (C) IL-6 and (D) TNFα was assessed by sandwich ELISA of cell supernatants. Data are shown as mean + SEM of three independent experiments, carried out in triplicates. Analyses were performed through the Mann-Whitney U Test. Comparisons with a p < 0.05 were considered significant. # Not detectable. (PDF) [file ppat.1006829.s008.pdf]

S9 Fig.

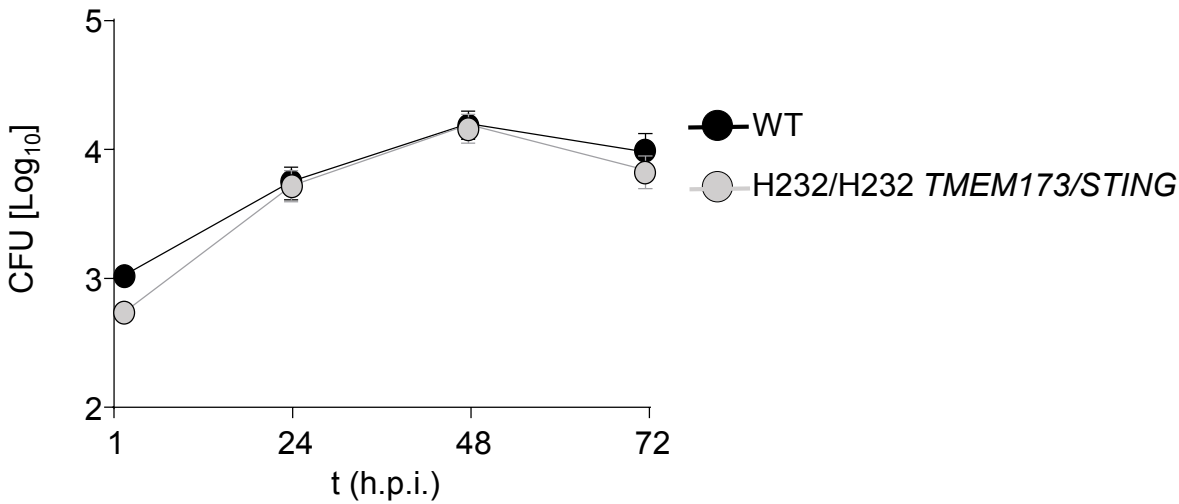

Supplement: S9 Fig — PBMCs from healthy volunteers (N = 3 for WT, N = 3 for R232H) were isolated by density gradient centrifugation. 7 d after isolation, cells were infected with L. pneumophila at MOI 0.1 and bacterial numbers were counted at the indicated time points. Data represent mean ± SEM of 3 independent experiments carried out in triplicates. (PDF) [file ppat.1006829.s009.pdf]
