# Supplementary material for: The common HAQ STING variant impairs cGAS-dependent antibacterial responses and is associated with susceptibility to Legionnaires’ disease in humans
Source: PLoS Pathog. 2018 Jan 3;14(1):e1006829. doi: 10.1371/journal.ppat.1006829 (PMC5770077; doi:10.1371/journal.ppat.1006829)
Supplement: S1 Table — (DOCX) [file ppat.1006829.s010.docx]

**Supplemental Table 1. Demographics of Netherlands cohort**

|  | Control  (N=88) | Cases  (N=91) |
| --- | --- | --- |
| Male, f*(n) | 0.5 (44) | 0.61 (56) |
| Age (median, IQR) | 49.6 (35.2 – 56.1) | 64.7 (54.2 – 71.5) |
| Smoker, f (n) | 0.31 (27) | 0.49 (45) |
| COPD, f (n) | 0.10 (8) | 0.07 (6) |
| Diabetes Mellitus, f (n) | 0.04 (3) | 0.09 (8) |
| Cancer, f(n) | 0.02 (2) | 0.04 (4) |
| Hx. Transplant, f(n) | 0.02 (2) | 0 (0) |
| Autoimmune Dz, f(n) | 0 (0) | 0.02 (2) |
| Alcohol Use, f(n) | 0.73 (61) | 0.35 (32) |

*f=frequency
